# Conditioned Pain Modulation Is Not Impaired in Individuals with Frozen Shoulder: A Case-Control Study

**DOI:** 10.3390/ijerph182312330

**Published:** 2021-11-24

**Authors:** Marta Aguilar-Rodríguez, Lirios Dueñas, Mercè Balasch i Bernat, Mira Meeus, Filip Struyf, Enrique Lluch

**Affiliations:** 1 Department of Physiotherapy, Faculty of Physiotherapy, University of Valencia, 46010 Valencia, Spain; marta.aguilar@uv.es (M.A.-R.); merce.balasch@uv.es (M.B.i.B.); enrique.lluch@uv.es (E.L.); 2 Physiotherapy in Motion, Multi Speciality Research Group (PTinMOTION), Department of Physiotherapy, Faculty of Physiotherapy, University of Valencia, 46010 Valencia, Spain; 3 Pain in Motion International Research Group, 2000 Antwerp, Belgium; mira.meeus@uantwerpen.be; 4 Department of Rehabilitation Sciences and Physiotherapy, Faculty of Medicine and Health Sciences, University of Antwerp, 2000 Antwerp, Belgium; filip.struyf@uantwerpen.be; 5 Department of Rehabilitation Sciences and Physiotherapy, Faculty of Medicine and Health Sciences, Ghent University, 9000 Ghent, Belgium; 6 Department of Physiotherapy, Human Physiology and Anatomy, Faculty of Physical Education & Physiotherapy, Vrije Universiteit Brussel, 1050 Brussels, Belgium

**Keywords:** frozen shoulder, conditioned pain modulation, inhibitory endogenous pain mechanisms, chronic pain, assessment

## Abstract

Frozen shoulder (FS) is a poorly understood condition resulting in substantial shoulder pain and mobility deficits. The mechanisms behind FS are not yet fully understood, but, similar to other persistent pain states, central pain mechanisms may contribute to ongoing symptoms in this population. The objective of this research was to investigate conditioned pain modulation (CPM) in people with FS compared with pain-free individuals. A total of 64 individuals with FS and 64 healthy volunteers participated in this cross-sectional study. CPM was assessed by using the pressure pain threshold (PPT) and an occlusion cuff (tourniquet test) as the test and conditioning stimulus, respectively. The absolute and percentage of change in PPT (CPM effect) as well as pain profiles (pro-nociceptive vs. anti-nociceptive) of individuals with FS and healthy controls were calculated. No significant differences in the absolute change in the PPT or CPM effect were found in people with FS compared to pain-free controls. Moreover, no between-group differences in the percentage of subjects with pro-nociceptive and anti-nociceptive pain profiles were observed. These results suggest that endogenous pain inhibition is normally functioning in people with FS. Altered central pain-processing mechanisms may thus not be a characteristic of this population.

## 1. Introduction

Frozen shoulder (FS), also known as adhesive capsulitis, is one of the most common yet challenging conditions for clinicians and physiotherapists. It is a poorly understood condition resulting in substantial shoulder pain and mobility deficits. The most notable clinical finding associated with FS is a global restriction of movement, with passive external rotation being the most limited direction [1]. The prevalence rate of FS in the general population is 2–5.3% [2,3], but it is higher (up to 40%) in individuals with diabetes [3].

Although FS has been widely studied, its epidemiology, etiology, diagnosis, and assessment are still poorly understood [4,5]. The pathophysiology of FS is characterized by an immune and inflammatory response and subsequent fibrosis of the anterior joint capsule and related structures (e.g., rotator cuff interval) [6,7]. The local inflammatory genesis of FS has been widely demonstrated both in animal [8] and human studies [9]. Moreover, research has shown, for instance, that local anesthetic suprascapular blocks improve pain and functionality, albeit for a short time, in people with FS [10,11]. All these findings would support a dominant role of peripheral mechanisms in the pain experienced by these individuals [12]. Indeed, conservative and surgical interventions have traditionally been focused on structural dysfunctions found around the shoulder joint, thus assuming pain related to FS as being predominantly dominated by peripheral mechanisms [13,14].

Recent evidence has questioned the contribution of peripheral mechanisms (i.e., capsular contracture) to movement restriction in some patients presenting with idiopathic FS [15]. Addtionally, some authors argue that central pain-processing mechanisms might play a role in a subgroup of people with FS [16]. Specifically, continuous nociceptive inputs in the early stages of FS may lead to maladaptive plastic reorganizations in different areas of the central nervous system (CNS) [16]. However, except for some recent studies [17,18,19], the role of the CNS (i.e., central sensitization (CS)) in these patients has not been fully studied and remains speculative. CS does not seem to be a characteristic feature of musculoskeletal shoulder pain but could be present in a subgroup of patients [20,21]. Acquiring further knowledge on pain mechanisms involved in FS is essential for designing better treatment strategies. For instance, if central pain-processing mechanisms are present in a subgroup of patients with FS, this could be one of the reasons that may explain why they are resistant to conventional peripherally focused therapies, and thus a different approach is required [16].

Different quantitative sensory testing tools, such as conditioned pain modulation (CPM), have been developed to quantify central pain mechanisms in people with musculoskeletal pain [22,23]. Historically, the term diffuse noxious inhibitory control (DNIC) was first proposed by Le Bars et al. [24] to describe the modulatory effect that a distant tonic painful conditioning stimulus has on a painful test stimulus. Later, in 2010, an expert panel recommended the use of the term CPM as a surrogate for estimating the DNIC phenomena in humans [25]. CPM thus represents a human assessment of the functionality of endogenous descending inhibitory pathways. CPM assessment can be helpful in predicting pain acquisition, in characterizing pain syndromes including dysfunctions of pain modulation, and in predicting response to treatments [26]. Regarding the latter, CPM has been shown to predict worse clinical outcomes, particularly the development of chronic post-operative pain, and the response to pharmacological interventions [27,28]. CPM can be impaired in several chronic pain populations, reflecting a decreased efficacy of endogenous pain inhibition [29]. Depending on CPM efficacy, individuals can be positioned on a clinical spectrum between pro-nociception or anti-nociception [30,31]. CPM effects have been studied in several chronic pain populations [32] but not yet in individuals with FS.

The main aim of this study was to examine the efficacy of CPM in individuals with FS compared to healthy controls. It was hypothesized that subjects with FS had impaired pain inhibition compared with healthy subjects. As a secondary aim, clinical pain profiles (i.e., pro-nociceptive vs. anti-nociceptive) of individuals with FS and healthy controls based on CPM results were compared.

## 2. Materials and Methods

### 2.1. Study Design

A case-control study following the STROBE guidelines [33] was carried out between July 2019 and July 2020 at the pain research unit of the University of Valencia (Valencia, Spain). The study was approved by the Human Research Ethics Committee of the University of Valencia (protocol number H1432625002427). All procedures were performed following the ethical standards laid down in the 1964 Declaration of Helsinki and its later amendments. Enrolled participants gave their informed consent prior to their inclusion in the study.

### 2.2. Participants

Eligible participants with primary FS from primary health care centers at Valencia Health Area provided written informed consent prior to participation in the study after FS had been diagnosed by their primary care doctor. Primary FS was diagnosed based on the following inclusion criteria: (1) more than 25% of range of motion loss in at least 2 planes of movement; (2) at least 50% of passive external rotation loss compared to the unaffected shoulder; and (3) presence of pain and restricted movement for at least 1 month, either having reached a peak or having worsened [1]. In addition, participants had to be stable in medication intake potentially influencing the results of CPM (e.g., anti-epileptic, antidepressant, analgesics, and/or NSAIDs) for at least 4 weeks prior to their participation to be included in this study [20]. Subjects with secondary FS (i.e., secondary to rotator cuff disease, trauma, or surgery) were excluded.

The control group consisted of pain-free and healthy subjects without a history of shoulder pain requiring medical treatment, matched by age and sex with the FS group [34]. Controls were recruited among university and hospital administrative staff.

### 2.3. Procedures

A trained physical therapist with 5 years of experience in assessing and treating individuals with FS and in the use of CPM was responsible for the assessments. This physical therapist remained blinded to group allocation. Participants were instructed not to reveal their diagnosis.

Participants’ socio-demographic information and pain intensity in the last 24 h using a visual analogue scale (VAS) was initially recorded [35]. Then, CPM was performed on each participant in a quiet temperature-controlled room with the subject seated in a relaxed position and with the non-affected arm supported on a table.

The protocol described by Cathcart and colleagues was adapted for measuring CPM [5]. First, the pressure pain threshold (PPT) was measured either at the middle deltoid (2 cm below the lateral part of the acromion) of the affected shoulder (in individuals with FS) or the matched shoulder (in healthy subjects) using a hand-held pressure algometer (Wagner Instruments, FDIX; Wagner Instruments, Greenwich, CT, USA) with a probe area of 1.0 cm^2^. The algometer probe tip was applied perpendicular to the skin and the pressure was increased at a rate of 1 kg/cm^2^/s until the point at which pressure sensation turned to pain [36]. Three measures were taken with a 30 s interval between each measurement, and the mean was used for analysis [37,38].

CPM was then induced by using an inflated occlusion cuff placed on the contralateral arm as the conditioned stimulus [5,39]. The occlusion cuff was inflated at a rate of 20 mm Hg/s until “the first sensation of pain”. At that point, it was maintained for 30 s. Pain intensity was then rated on a numerical pain rating scale. Next, cuff inflation was increased or decreased until the pain intensity was rated as 3/10. PPT assessment was then repeated during maintenance of the cuff inflation [40].

### 2.4. Outcomes

The absolute change and the percentage of change (or CPM effect) were measured as recommended by Kennedy et al. [41] and Yarnitsky et al. [42]. The absolute change was calculated as the difference between the PPT during the conditioned stimulus and the PPT before the conditioned stimulus. The percentage of change (or the CPM effect) was calculated using the following formula [43]:

(1) $$\mathrm{CPM}\;\mathrm{effect}=\frac{\mathrm{PPT}\;\mathrm{during}\;\mathrm{CSt}\;-\mathrm{PPT}\;\mathrm{before}\;\mathrm{CSt}}{\mathrm{PPT}\;\mathrm{before}\;\mathrm{CSt}}\times 100$$

where CPM denotes the conditioned pain modulation, CSt denotes the conditioned stimulus, and PPT denotes the pressure pain threshold.

### 2.5. Statistical Analysis

Descriptive statistics were used to present demographic and clinical information. Data normality was assessed using the Kolmogorov–Smirnoff test. Data results are expressed as the mean (standard deviation) in case of continuous variables and relative and absolute frequencies in case of categorical variables. Independent *t*-tests were used to compare absolute change and CPM effect between participants with FS and healthy controls.

The percentage of individuals with FS and healthy controls presenting a pro-nociceptive profile or inefficient CPM and an anti-nociceptive profile or efficient CPM [42,44] was calculated based on the inherent measurement error [31]. Those individuals in whom the CPM effect was ≤5.3% were classified as pro-nociceptive, whereas a CPM effect of >5.3% was considered as anti-nociceptive [30,42]. Statistical significance was set at a *p*-value of <0.05. The chi-squared test was used to determine whether there were statistically significant between-group differences regarding the pain profile.

## 3. Results

A total of 64 individuals with FS were age-, sex-, and side-matched with 64 healthy control subjects. The demographic data of the study participants are summarized in Table 1.

Statistically significant differences were found between individuals with FS and healthy controls in weight. In addition, individuals with FS showed a mean basal PPT significantly lower than healthy subjects (FS: mean = 320.63, SD = 197.39 kPa; healthy: mean = 600.95, SD = 266.16 kPa; *p* = 0.00).

There were no significant between-group differences neither in the absolute change nor in the CPM effect (Table 2).

A total of 24 subjects with FS (37.50%) showed a pro-nociceptive profile or an inefficient CPM, and 40 (62.50%) showed an anti-nociceptive profile or an efficient CPM. Among healthy subjects, 23 (35.93%) showed a pro-nociceptive profile, and 41 (64.06%) showed an anti-nociceptive profile. There were no significant between-group differences in the percentage of individuals presenting a pro-nociceptive profile and an anti-nociceptive profile (*χ*^2^: 0.034, *p* > 0.05).

## 4. Discussion

This study investigated the efficiency of inhibitory endogenous pain mechanisms in subjects with FS by using the CPM paradigm. The results showed that subjects with FS do not present altered CPM efficacy compared to healthy controls, as no significant differences in the absolute change or the CPM effect were found. In addition, no significant differences in the clinical pain profiles between subjects with FS and healthy controls were found as reflected by the similar percentage of individuals showing both pro-nociceptive and anti-nociceptive profiles in both groups. These findings contradict our initial hypothesis and suggest that impaired pain inhibitory mechanisms might not contribute to the pain experienced by individuals with FS, as shown in other chronic pain conditions.

Our results are contrary to previous reviews that showed impaired CPM in people with chronic pain in comparison topain-free controls [29]. However, recent studies that assessed CPM in some chronic pain populations (i.e., painful diabetic neuropathy [30] or temporomandibular disorders [45]) did not find differences in the CPM response between patients and controls. Granovsky et al. [30] argued that this lack of between-group differences in pain inhibition might be due to an effort of the CNS-pain-controlling subsystems to return to equilibrium, even when the perturbing factor (i.e., pain) is still prevalent. In our study, the mean duration of symptoms of individuals with FS (i.e., 7 months) might have been enough to induce the mentioned equilibrium between groups in pain-controlling subsystems.

Over the last years, abnormal CPM has been considered a valid biomarker of chronic pain [46]. However, a recent systematic review questioned the clinical value of CPM as the majority of studies analyzed reported non-significant correlations between CPM efficiency and clinical manifestations of pain [23]. Heterogeneity in the methodology employed among studies was highlighted by the authors as the main reason explaining those results. In this sense, the methodology we used to measure CPM was very similar to recent recommendations by Vaegter et al. [47], who found that using PPT as the test stimulus and occlusion cuff as the conditioning stimulus was the most reliable among 10 different paradigms for assessing CPM. However, besides methodology, other factors have been shown to influence the CPM response, such as oral contraceptives, catastrophizing, information about conditioning stimulation, distraction, physical activity, or genetics [48,49]. These factors were not taken into account in our study and might have influenced our CPM results.

Although patients with FS did not present an inefficient CPM compared to healthy controls at an average population level, we cannot rule out that altered central pain-processing mechanisms might be present at an individual level [50]. Indeed, an individual assessment has been recommended to detect altered pain processing. In addition, we only measured one surrogate of central hypersensitivity (i.e., CPM), so future research could measure other suggested indices of CS, such as the temporal summation or the nociceptive flexion reflex in individuals with FS, to evaluate if there are differences in pain-processing mechanisms in comparison to healthy subjects.

Applying a painful treatment can inhibit the patient’s pain due to the phenomenon of counter-irritation-induced analgesia, which is mediated by descending inhibitory pathways [4]. The fact that CPM was not altered in our study may imply that individuals with FS would maintain their ability to inhibit pain when receiving a painful stimulus, for example, in the form of manual therapy or exercises. It has been argued that the application of painful treatment techniques in subjects with FS may be counterproductive and could even lead to poor therapeutic outcomes [51,52]. In line with this, recent studies have identified a relationship between the presence of dominant central pain-processing mechanisms and poor response to physical therapy interventions [53]. According to our results, the mechanism by which painful treatment techniques could aggravate the patient’s condition or explain poor outcomes after treatment in individuals with FS would not be explained by impairment in CPM. It would be interesting, for instance, to investigate whether treatment responses are different in subgroups of individuals with FS with pro-nociceptive versus anti-nociceptive profiles.

Consideration must be given to the limitations of this study. Despite having insufficient data to identify a certain CPM protocol as preferable, it has been recommended in the literature to use two types of test stimuli (thermal and mechanical) [46]. We only used a mechanical stimulus in this study. In addition, a parallel protocol (i.e., presenting the conditioned stimulus in parallel to the conditioning stimulus) was used instead of a sequential protocol, as recommended by expert opinion [46]. The examiner performing the CPM assessment was blinded to the group allocation and participants were instructed not to reveal their diagnosis. However, it cannot be discarded that during the CPM procedure, the case group demonstrated a greater functional limitation, thus negatively influencing the blinding process.

## 5. Conclusions

The present results suggest that subjects with FS do not exhibit a loss of descending pain inhibition (i.e., impaired CPM) compared to healthy controls. Thus, alteration in central pain-processing mechanisms seems to not be a characteristic of subjects with FS. However, further research using other indices of CS is needed to draw firm conclusions.

## Figures and Tables

**Table 1:** Basal descriptive data.

	FS (*n* = 64)	Healthy (*n* = 64)
Age (years)	53.58 (7.65)	52.63 (6.81)
Height (cm)	165.47 (1.06)	166.63 (0.90)
Weight (kg)	65.16 (1.49)	69.66 (1.35) ^1^
Gender:		
Male	20 (31.25%)	20 (31.25%)
Female	44 (68.75%)	44 (68.75%)
Affected or measured side:		
Left	30 (46.88%)	30 (46.88%)
Right	34 (53.12%)	34 (53.12%)
Duration of symptoms (months)	7.36 (4.21)	-

Data are expressed as mean (standard deviation) or *n* (frequency). FS: frozen shoulder; SD, standard deviation. ^1^ *p* < 0.05.

**Table 2:** Pressure pain thresholds, absolute change, and conditioned pain modulation effect values in individuals with frozen shoulder and healthy controls.

	FS (*n* = 64)	Healthy (*n* = 64)
Basal PPT (Kpa)	320.63 (197.39)	600.95 (266.16) ^1^
Absolute change (kPa)	28.51 (92.71)	67.01 (149.25)
CPM Effect (%)	12.42 (29.87)	14.29 (25.02)

Data are expressed as mean (standard deviation) or *n* (frequency). FS: frozen shoulder; SD, standard deviation; PPT, pressure pain threshold. ^1^ *p* < 0.05.

## Data Availability

Not available.

## References

[1] Kelley M.J., Shaffer M.A., Kuhn J.E., Michener L.A., Seitz A.L., Uhl T.L., Godges J.J., McClure P.W. (2013). Shoulder Pain and Mobility Deficits: Adhesive Capsulitis. J. Orthop. Sports Phys. Ther..

[2] Neviaser A.S., Hannafin J.A. (2010). Adhesive Capsulitis: A Review of Current Treatment. Am. J. Sports Med..

[3] Tasto J.P., Elias D.W. (2007). Adhesive Capsulitis. Sports Med. Arthrosc. Rev..

[4] Carlton S.M., Zhou S., Kraemer B., Coggeshall R.E. (2003). A Role for Peripheral Somatostatin Receptors in Counter-Irritation-Induced Analgesia. Neuroscience.

[5] Cathcart S., Winefield A.H., Lushington K., Rolan P. (2010). Noxious Inhibition of Temporal Summation Is Impaired in Chronic Tension-Type Headache. Headache.

[6] Akbar M., McLean M., Garcia-Melchor E., Crowe L.A., McMillan P., Fazzi U.G., Martin D., Arthur A., Reilly J.H., McInnes I.B. (2019). Fibroblast Activation and Inflammation in Frozen Shoulder. PLoS ONE.

[7] Andronic O., Ernstbrunner L., Jüngel A., Wieser K., Bouaicha S. (2020). Biomarkers Associated with Idiopathic Frozen Shoulder: A Systematic Review. Connect. Tissue Res..

[8] Kim D.H., Lee K.-H., Lho Y.-M., Ha E., Hwang I., Song K.-S., Cho C.-H. (2016). Characterization of a Frozen Shoulder Model Using Immobilization in Rats. J. Orthop. Surg..

[9] Hand G.C.R., Athanasou N.A., Matthews T., Carr A.J. (2007). The Pathology of Frozen Shoulder. J. Bone Joint Surg. Br..

[10] Karataş G.K., Meray J. (2002). Suprascapular Nerve Block for Pain Relief in Adhesive Capsulitis: Comparison of 2 Different Techniques. Arch. Phys. Med. Rehabil..

[11] Bae K.H., Park K.C., Jeong G.M., Lim T.K. (2021). Proximal vs Distal Approach of Ultrasound-Guided Suprascapular Nerve Block for Patients With Adhesive Capsulitis of the Shoulder: Prospective Randomized Controlled Trial. Arch. Phys. Med. Rehabil..

[12] Dean B.J.F., Gwilym S.E., Carr A.J. (2013). Why Does My Shoulder Hurt? A Review of the Neuroanatomical and Biochemical Basis of Shoulder Pain. Br. J. Sports Med..

[13] Lowe C.M., Barrett E., McCreesh K., De Búrca N., Lewis J. (2019). Clinical Effectiveness of Non-Surgical Interventions for Primary Frozen Shoulder: A Systematic Review. J. Rehabil. Med..

[14] Le H.V., Lee S.J., Nazarian A., Rodriguez E.K. (2017). Adhesive Capsulitis of the Shoulder: Review of Pathophysiology and Current Clinical Treatments. Shoulder Elb..

[15] Hollmann L., Halaki M., Kamper S.J., Haber M., Ginn K.A. (2018). Does Muscle Guarding Play a Role in Range of Motion Loss in Patients with Frozen Shoulder?. Musculoskelet. Sci. Pract..

[16] Struyf F., Meeus M. (2014). Current Evidence on Physical Therapy in Patients with Adhesive Capsulitis: What Are We Missing?. Clin. Rheumatol..

[17] Mena-del Horno S., Balasch-Bernat M., Dueñas L., Reis F., Louw A., Lluch E. (2020). Laterality Judgement and Tactile Acuity in Patients with Frozen Shoulder: A Cross-Sectional Study. Musculoskelet. Sci. Pract..

[18] Breckenridge J.D., McAuley J.H., Ginn K.A. (2020). Motor Imagery Performance and Tactile Spatial Acuity: Are They Altered in People with Frozen Shoulder?. Int. J. Environ. Res. Public. Health.

[19] Louw A., Puentedura E.J., Reese D., Parker P., Miller T., Mintken P.E. (2017). Immediate Effects of Mirror Therapy in Patients With Shoulder Pain and Decreased Range of Motion. Arch. Phys. Med. Rehabil..

[20] Kuppens K., Hans G., Roussel N., Struyf F., Fransen E., Cras P., Van Wilgen C.P., Nijs J. (2018). Sensory Processing and Central Pain Modulation in Patients with Chronic Shoulder Pain: A Case-Control Study. Scand. J. Med. Sci. Sports.

[21] Haik M.N., Evans K., Smith A., Henríquez L., Bisset L. (2019). People with Musculoskeletal Shoulder Pain Demonstrate No Signs of Altered Pain Processing. Musculoskelet. Sci. Pract..

[22] Arendt-Nielsen L., Morlion B., Perrot S., Dahan A., Dickenson A., Kress H.G., Wells C., Bouhassira D., Mohr Drewes A. (2018). Assessment and Manifestation of Central Sensitisation across Different Chronic Pain Conditions. Eur. J. Pain Lond. Engl..

[23] Fernandes C., Pidal-Miranda M., Samartin-Veiga N., Carrillo-de-la-Peña M.T. (2019). Conditioned Pain Modulation as Biomarker of Chronic Pain: A Systematic Review of Its Concurrent Validity. Pain.

[24] Le Bars D., Dickenson A.H., Besson J.-M. (1979). Diffuse Noxious Inhibitory Controls (DNIC). I. Effects on Dorsal Horn Convergent Neurones in the Rat. Pain.

[25] Yarnitsky D., Arendt-Nielsen L., Bouhassira D., Edwards R.R., Fillingim R.B., Granot M., Hansson P., Lautenbacher S., Marchand S., Wilder-Smith O. (2010). Recommendations on Terminology and Practice of Psychophysical DNIC Testing. Eur. J. Pain.

[26] Yarnitsky D. (2015). Role of Endogenous Pain Modulation in Chronic Pain Mechanisms and Treatment. Pain.

[27] Petersen K.K., Vaegter H.B., Stubhaug A., Wolff A., Scammell B.E., Arendt-Nielsen L., Larsen D.B. (2021). The Predictive Value of Quantitative Sensory Testing: A Systematic Review on Chronic Postoperative Pain and the Analgesic Effect of Pharmacological Therapies in Patients with Chronic Pain. Pain.

[28] Georgopoulos V., Akin-Akinyosoye K., Zhang W., McWilliams D.F., Hendrick P., Walsh D.A. (2019). Quantitative Sensory Testing and Predicting Outcomes for Musculoskeletal Pain, Disability, and Negative Affect: A Systematic Review and Meta-Analysis. Pain.

[29] Lewis G.N., Rice D.A., McNair P.J. (2012). Conditioned Pain Modulation in Populations with Chronic Pain: A Systematic Review and Meta-Analysis. J. Pain Off. J. Am. Pain Soc..

[30] Granovsky Y., Nahman-Averbuch H., Khamaisi M., Granot M. (2017). Efficient Conditioned Pain Modulation despite Pain Persistence in Painful Diabetic Neuropathy. PAIN Rep..

[31] Locke D., Gibson W., Moss P., Munyard K., Mamotte C., Wright A. (2014). Analysis of Meaningful Conditioned Pain Modulation Effect in a Pain-Free Adult Population. J. Pain Off. J. Am. Pain Soc..

[32] Petersen K.K., McPhee M.E., Hoegh M.S., Graven-Nielsen T. (2019). Assessment of Conditioned Pain Modulation in Healthy Participants and Patients with Chronic Pain: Manifestations and Implications for Pain Progression. Curr. Opin. Support. Palliat. Care.

[33] von Elm E., Altman D.G., Egger M., Pocock S.J., Gøtzsche P.C., Vandenbroucke J.P. (2007). The Strengthening the Reporting of Observational Studies in Epidemiology (STROBE) Statement: Guidelines for Reporting Observational Studies. Epidemiology.

[34] Ravat S., Olivier B., Gillion N., Lewis F. (2018). Laterality Judgment Performance between People with Chronic Pain and Pain-Free Individuals: A Systematic Review Protocol. JBI Database Syst. Rev. Implement. Rep..

[35] Bijur P.E., Silver W., Gallagher E.J. (2001). Reliability of the Visual Analog Scale for Measurement of Acute Pain. Acad. Emerg. Med. Off. J. Soc. Acad. Emerg. Med..

[36] Banic B., Petersen-Felix S., Andersen O.K., Radanov B.P., Villiger P.M., Arendt-Nielsen L., Curatolo M. (2004). Evidence for Spinal Cord Hypersensitivity in Chronic Pain after Whiplash Injury and in Fibromyalgia. Pain.

[37] Slater H., Arendt-Nielsen L., Wright A., Graven-Nielsen T. (2005). Sensory and Motor Effects of Experimental Muscle Pain in Patients with Lateral Epicondylalgia and Controls with Delayed Onset Muscle Soreness. Pain.

[38] Slater H., Thériault E., Ronningen B.O., Clark R., Nosaka K. (2010). Exercise-Induced Mechanical Hypoalgesia in Musculotendinous Tissues of the Lateral Elbow. Man. Ther..

[39] Fujii K., Motohashi K., Umino M. (2006). Heterotopic Ischemic Pain Attenuates Somatosensory Evoked Potentials Induced by Electrical Tooth Stimulation: Diffuse Noxious Inhibitory Controls in the Trigeminal Nerve Territory. Eur. J. Pain Lond. Engl..

[40] Cathcart S., Winefield A.H., Rolan P., Lushington K. (2009). Reliability of Temporal Summation and Diffuse Noxious Inhibitory Control. Pain Res. Manag..

[41] Kennedy D.L., Kemp H.I., Ridout D., Yarnitsky D., Rice A.S.C. (2016). Reliability of Conditioned Pain Modulation: A Systematic Review. Pain.

[42] Yarnitsky D., Granot M., Granovsky Y. (2014). Pain Modulation Profile and Pain Therapy: Between pro- and Antinociception. Pain.

[43] Valencia C., Kindler L.L., Fillingim R.B., George S.Z. (2013). Stability of Conditioned Pain Modulation in Two Musculoskeletal Pain Models: Investigating the Influence of Shoulder Pain Intensity and Gender. BMC Musculoskelet. Disord..

[44] Granovsky Y., Miller-Barmak A., Goldstein O., Sprecher E., Yarnitsky D. (2016). CPM Test-Retest Reliability: “Standard” vs. “Single Test-Stimulus” Protocols. Pain Med. Malden Mass.

[45] Moana-Filho E.J., Herrero Babiloni A. (2019). Endogenous Pain Modulation in Chronic Temporomandibular Disorders: Derivation of Pain Modulation Profiles and Assessment of Its Relationship with Clinical Characteristics. J. Oral Rehabil..

[46] Yarnitsky D., Bouhassira D., Drewes A.M., Fillingim R.B., Granot M., Hansson P., Landau R., Marchand S., Matre D., Nilsen K.B. (2015). Recommendations on Practice of Conditioned Pain Modulation (CPM) Testing. Eur. J. Pain Lond. Engl..

[47] Vaegter H.B., Petersen K.K., Mørch C.D., Imai Y., Arendt-Nielsen L. (2018). Assessment of CPM Reliability: Quantification of the within-Subject Reliability of 10 Different Protocols. Scand. J. Pain.

[48] Hermans L., Van Oosterwijck J., Goubert D., Goudman L., Crombez G., Calders P., Meeus M. (2016). Inventory of Personal Factors Influencing Conditioned Pain Modulation in Healthy People: A Systematic Literature Review. Pain Pract..

[49] Goubert D., Danneels L., Cagnie B., Van Oosterwijck J., Kolba K., Noyez H., Meeus M. (2015). Effect of Pain Induction or Pain Reduction on Conditioned Pain Modulation in Adults: A Systematic Review. Pain Pract. Off. J. World Inst. Pain.

[50] Schliessbach J., Siegenthaler A., Streitberger K., Eichenberger U., Nüesch E., Jüni P., Arendt-Nielsen L., Curatolo M. (2013). The Prevalence of Widespread Central Hypersensitivity in Chronic Pain Patients. Eur. J. Pain.

[51] Dupeyron A., Dénarié M., Richard D., Dobija L., Castelli C., Petiot S., Tavares I., Gelis A., Coudeyre E. (2019). Analgesic Gas for Rehabilitation of Frozen Shoulder: Protocol for a Randomized Controlled Trial. Ann. Phys. Rehabil. Med..

[52] Diercks R.L., Stevens M. (2004). Gentle Thawing of the Frozen Shoulder: A Prospective Study of Supervised Neglect versus Intensive Physical Therapy in Seventy-Seven Patients with Frozen Shoulder Syndrome Followed up for Two Years. J. Shoulder Elbow Surg..

[53] O’Leary H., Smart K.M., Moloney N.A., Blake C., Doody C.M. (2018). Pain Sensitization Associated with Nonresponse after Physiotherapy in People with Knee Osteoarthritis. Pain.

